# Optimization of re-irradiation using deformable registration: a case study

**DOI:** 10.1259/bjrcr.20150412

**Published:** 2016-05-06

**Authors:** Raphaël Jumeau, Nicolas Péguret, Cédric Zulliger, Raphaël Moeckli, Jean Bourhis, Esat-Mahmut Ozsahin

**Affiliations:** ^1^ Department of Radiation Oncology, Centre Hospitalier Universitaire Vaudois (CHUV) and University of Lausanne, Lausanne, Switzerland; ^2^ Institute of Radiation Physics, Centre Hospitalier Universitaire Vaudois (CHUV) and University of Lausanne, Lausanne, Switzerland

## Abstract

Re-irradiation is frequently performed in radiotherapy (RT) departments. We present an optimization methodology that takes the previous irradiation into account. A 68-year-old female patient suffering from rectal adenocarcinoma, who had previously undergone RT for metastases to the right iliac bone, presented with a recurrence of metastasis to the L5 and the left sacroiliac joint. Re-irradiation was performed using volumetric modulated arc therapy (VMAT). We proceeded to a registration of the previous RT planning CT and RT doses to the new planning CT. Virtual volumes corresponding to the intersection of the small bowel (SB) and each isodose structure were created. We calculated the maximal dose (D_max_) that each virtual structure could receive and considered them as constraints. We called this technique modified VMAT. We compared this technique with a standard VMAT plan and a three-dimensional RT plan. Using the modified VMAT technique, a total dose of 20 Gy in five fractions of 4 Gy was delivered to the planning target volume without any acute toxicity. A composite dosimetry was realized with each technique to compare the dose given to the already irradiated SB. We calculated the D_max_ received by the already irradiated SB in equivalent dose of 2 Gy fractions. The Dmax was 46.8, 60 and 52 Gy for modified VMAT, standard VMAT and three-dimensional RT, respectively. Dose deformation was used to create new constraint structures to optimize the dose delivered to surrounding tissues. This methodology is readily feasible in clinical routine to optimize the re-irradiation process.

## Introduction

Thanks to new treatment techniques in radiotherapy (RT) and oncology, patients suffering from cancer live longer than before.^1^ Nowadays, we frequently observe patients who need several courses of RT.^2^ Sometimes, re-irradiation is needed in the same area that had previously been treated. Intersection with the previous treatment area is a reason to reduce the dose in order to not exceed a critical dose appropriate for each organ. The critical issue in the re-irradiation setting, dealing not only with palliative patients but also those with curative intent, is to avoid side effects in the surrounding normal organs. Therefore, the dose delivered to the target is often compromised. Intensity-modulated radiotherapy (IMRT) has been a major advancement in RT, improving critical organ sparing by using the concept of inverse planning. More recently, deformable image registration (DIR), used to warp an image on another, has been integrated into many steps of the RT process.^3^ The goal of this study is to present a new methodology based on DIR that takes into account the previous irradiation in order to optimize the re-irradiation plan.

## Materials and methods

### Patient

This new concept will be described on a single patient. A 68-year-old female patient suffering from rectal adenocarcinoma with initial multimetastatic evolution (liver, bones and lung) since September 2013 was referred. She underwent a first course of RT for hyperalgic bone metastases to the seventh cervical vertebra, left shoulder, right iliac bone and right femoral articulation towards the end of 2013. A total dose of 30 Gy in 10 fractions of 3 Gy five times a week was delivered to the right iliac bone and the right femoral articulation. A three-dimensional conformal RT (3D RT) technique was used to perform this treatment. Following RT, she was offered palliative chemotherapy with FOLFIRI (folinic acid, fluorouracil and irinotecan) and bevacizumab. In April 2014, she presented with recurrence of pain that was localized to the front of the fifth lumbar vertebra. A CT scan confirmed metastatic infiltration of the L5 and the left sacroiliac joint.

### Methodology

#### Simulation

In order to reproduce the initial set-up, the patient was positioned supine on a thin foam mattress with a foam support under the knees, in accordance with the initial planning CT scan. CT scan was performed with a Toshiba Aquilion LB^®^ (Toshiba Medical Systems, Zoetermeer, Netherlands), with an image thickness of 2 mm as in the first planning CT. CT acquisition was made to cover the whole pevis area and the lumbar spine.

#### Target and organs at risk definition

The clinical target volume corresponded to the L5 vertebra and the left sacroiliac joint. The planning target volume (PTV) was created by a 5 mm automatic expansion of the clinical target volume. The small bowel (SB), which was the most critical organ of this area and had already been irradiated, was delineated with respect to the peritoneal cavity.^4^ Structures and registration were performed using VelocityAI 3.1.0^®^ software (Velocity Medical Solutions, Atlanta, GA). We first rigidly aligned the previous treatment planning CT (CT1) on the new planning CT (CT2) according to the bony structures. Then, a DIR between CT1 and CT2 was performed to create a transformation that was applied on the previous dose to fit with CT2. The quality of deformable registration was visually checked by the physician. Once the previous dose had been deformed on CT2, an automatic delineation of isodoses was performed. An isodose was created for every 5 Gy interval. In order to define different constraint volumes, we determined a virtual volume corresponding to the intersection of SB and every isodose structure and called it OARsbX (X representing the corresponding isodose). We obtained six virtual structures. Each of these structures represented the estimated part of the SB that had already received a part of the previous irradiation. The volume corresponding to the intersection between the 5 Gy isodose and the SB was defined as the already irradiated SB (AISB).

#### Treatment planning

The new RT plan consisted of a volumetric modulated arc therapy (VMAT) treatment. An inverse dosimetry plan was performed using the Monaco treatment planning system (TPS) (Elekta Instrument AB, Stockholm, Sweden). Owing to the previous treatment being hypofractionated RT, each of the OARsbX was computed in equivalent dose of 2 Gy fractions (EQD2).^5,6^ We chose an α/β ratio of 2 Gy for the SB.^7^ The Radiation Therapy Oncology Group 0822^8^ study constraints were used to determine the appropriate maximal dose (D_max_) to deliver to each of the OARsbX without exceeding the D_max_ of 50 Gy. OARsbXs were used as constraint volumes, where D_max_ = 50 – OARsbX_EQD2_. We calculated the D_max_ that each OARsbX could receive and entered them into the TPS as constraints. This methodology was designed to optimize the new treatment integrating the previous treatment. This technique was named modified VMAT.

#### Comparison

VMAT is used worldwide and is well known to reduce gastrointestinal toxicity.^9,10^ 3D RT could be a simple, fast and efficient treatment that completely avoids the AISB. For comparison, we also computed a 3D RT and a VMAT plan without using OARsbX constraints. For each plan, we measured the D_max_ and the minimum dose (D_min_) within the target volume.^11^ We compared the three re-irradiation plans and also performed three composite dosimetries of the first and second irradiation to underline the best technique that would preserve the AISB and reduce the risk of radiation-induced diseases.^12^ The most used constraint was V45 < 195 cc according to Quantitative Analyses of Normal Tissue Effects in the Clinic (QUANTEC) for SB,^13^ and this was also studied during the plan comparison. Treatments were not given at the same regimen of doses; therefore, we calculated the equivalent V45 on each composite dosimetry in two different ways: if the whole treatment was given in fractions of 3 Gy or 4 Gy.

## Results

### Delivered treatment

A total dose of 20 Gy in five fractions of 4 Gy was delivered to the PTV in 1 week without any acute gastrointestinal toxicity. The results are presented in Table 1 a.

**Table 1.** Dosimetric results of the second treatment with different techniques and the second treatment and composite values

	VMAT*	VMAT	3D RT
**(a) *Second treatment only***			
**PTV**			
D95 (Gy)	18.6	18.9	15
**SB**			
D_max_(Gy)	21	20.7	21.5
**AISB**			
D_max_(Gy)	18.6	20	14.9
Conformity index (TV/PTV)	0.89	0.88	0.35
**(b) *Composite dosimetry***			
**SB**			
V30_EQD2 _(cc)	312.3	312.7	97.5
V36_EQD2_ (cc)	4.3	50	1.6
**AISB**			
D_max_(Gy)	39.5	47.9	43.1
D_max_ EQD2 (Gy)	46.8	60	52

3D RT, three-dimensional conformal radiotherapy; AISB, already irradiated small bowel; D_max_, maximum dose; EQD2, dose equivalent in 2 Gy per fraction; PTV, planning treatment volume; SB, small bowel; TV, treated volume; VMAT, volumetric modulated arc therapy; VMAT*, modified volumetric modulated arc therapy.

### Plan comparison

**Figure 1.**
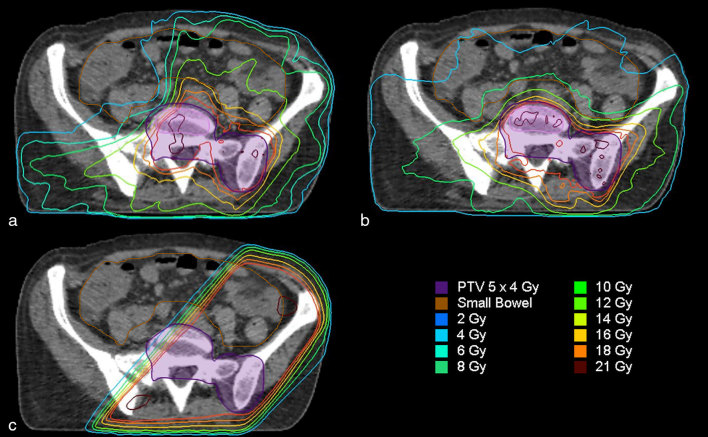
Radiotherapy plan of each technique tested: (a) VMAT*; (b) VMAT; and (c) 3D RT. 3D RT, three-dimensional conformal radiotherapy; PTV, planning treatment volume; VMAT, volumetric modulated arc therapy; VMAT*, modified volumetric modulated arc therapy.

The RT plans are presented in Figure 1. We compared the conformity index^14^ of the PTV for each technique. We compared the dose received by the SB and the AISB with each technique. The results are presented in Table 1 a.

### Composite dosimetry comparison

V45 was converted to EQD2, with an α/β ratio of 2 Gy for the SB. In 3 Gy fractions, V45 corresponded to V36_EQD2_, and in 4 Gy fractions, it corresponded to V30_EQD2_. The D_max_ of AISB and V45 of SB were compared for each technique and the results are presented in Table 1 b.

## Discussion

Oncology now deals with cancer patients with prolonged survival and radiologists are increasingly being solicited to perform re-irradiation. The use of re-irradiation is limited by the risks of complications and there is a need for dose/volume optimization, especially with high doses in curative treatments. DIR is a very interesting technology for multimodality image fusion, dose accumulation or anatomic image segmentation.^15^ Dose warping using DIR is useful in evaluating the dose accumulation between previous and current RT plans for re-irradiated patients. With IMRT, the concept of inverse planning allows us to spare virtual structures as OAR. Our methodology using deformable registration by creating virtual constraint volumes represents an interesting alternative to the usual techniques to secure re-irradiation.

It must be pointed out that, in this study, we used dose deformation to create new constraint structures. However, in this case, the DIR software also allows to proceed to cumulative dosimetry^16^ in order to better appreciate the re-irradiation parameters. For that reason, during cumulative dosimetry, some uncertainties have to be taken into account. In fact, rigid deformation is more accurate than deformable registration^17–19^ and several anatomic changes can disturb the accuracy of the registration. Nonetheless, the pelvis is probably the best area to proceed with deformable registration, as the sacroiliac bones help in controlling the accuracy of the registration. Deformable registration is not recommended in case of major anatomic changes to avoid errors.

In the case described previously, we showed the efficiency of our technique. The major interest of this technique is in securing re-irradiation and increasing its clinical efficacy. With the use of this technique, the TPS can be forced to avoid a high-risk zone with controlled dose degradation to the PTV. In addition, modified VMAT was the only technique that did not exceed 50 Gy as D_max_ and was the best technique for most dosimetric parameters. At final verification, the physician should consider three RT plans: the first treatment, the new treatment plan and the composite dosimetry. Each element gives information, and, therefore, cumulative dosimetry should not be the only element of decision-making because of the risk of errors related to deformable registration.

A limitation of this study was the impossibility to use the composite dose–volume histogram value to determine the D_max_ because of the use of different fractionation regimens. For the same reason, the exact V45 value could not be determined. A case without fractionation modification would be more convenient to appreciate these parameters. In the most pessimistic scenario, V30_EQD2_ was superior to 195 cc but it is overestimated. In fact, most of the treatment was given in fractions of 3 Gy. Then, the most realistic value of V45 was closer to V36_EQD2_ than V30_EQD2_, which was acceptable with all techniques. Finally, it could be of interest to evaluate the duration of this procedure to show that it does not extend the planning process.

## Conclusions

In conclusion, we presented a methodology for re-irradiation that takes the previous irradiation into account and optimizes the dose delivered to the patient. There is no practical difficulty for the medical teams who are familiar with IMRT techniques and have access to DIR software to use this technique. Thanks to DIR, we can perhaps offer re-irradiation more often and deliver safer treatment to patients.

## Consent

Informed consent was obtained and held on medical records. Patient data has been anonymized.
